# The study on the outsourcing of Taiwan's hospitals: a questionnaire survey research

**DOI:** 10.1186/1472-6963-9-78

**Published:** 2009-05-13

**Authors:** Chih-Tung Hsiao, Jar-Yuan Pai, Hero Chiu

**Affiliations:** 1 Department of Economics, Tunghai University, Taichung 40704, Taiwan, R.O.C.; 2 Department of Healthcare Services Administration, Chung Shan Medical University, Taichung 402, Taiwan, R.O.C.; 3 Chung Shan Medical University Hospital, Taichung 40201, Taiwan, R.O.C.; 4 Department of Counseling and Applied Psychology, National Taichung University, Taichung 40306, Taiwan, R.O.C.

## Abstract

**Background:**

The aim of this study was to assess the outsourcing situation in Taiwanese hospitals and compares the differences in hospital ownership and in accreditation levels.

**Methods:**

This research combined two kinds of methods: a questionnaire survey and the in-depth interview to two CEOs of the sample hospitals. One hospital is not-for-profit, while the other is a public hospital and the research samples are from the hospital data from Taiwan's 2005 to 2007 Department of Health qualifying lists of hospital accreditation. The returned questionnaires were analyzed with STATISTICA^® ^7.1 version software.

**Results:**

The results for non-medical items showed medical waste and common trash both have the highest rate (94.6 percent) of being outsourced. The gift store (75 percent) and linen (73 percent) follow close behind, while the lowest rate of outsourcing is in utility maintenance (13.5 percent). For medical items, the highest rate of outsourcing is in the ambulance units (51.4 percent), while the hemodialysis center follows close behind with a rate of 50 percent. For departments of nutrition, pharmacy, and nursing however, the outsourcing rate is lower than 3 percent. This shows that Taiwan's hospitals are still conservative in their willingness to outsource for medical items. The results of the satisfaction paired t-test show that the non-medical items have a higher score than the medical items. The factor analysis showed the three significant factors in of non medical items' outsourcing are "performance", "finance", and "human resource". For medical items, the two factors are "operation" and satisfaction". To further exam the factor validity and reliability of the satisfaction model, a confirmative factor analysis (CFA) was conducted using structure equation modeling (SEM) method and found the model fitting well.

**Conclusion:**

Hospitals, especially for public hospitals, can get benefits from outsourcing to revive the full-time-equivalent and human resource limitation.

## Background

According to Modern Healthcare's 27th Annual Outsourcing Survey in the USA, the number of outsourcing contracts for respondents continues to rise. The 20 largest outsourcing companies reported a combined number of 11,324 healthcare clients, which is up 10.6 percent from the previous year. Laundry jumped ahead of housekeeping as the top hospital department-management contract in this year's annual Outsourcing Survey, with a total of 4,443 contracts in 2004. Housekeeping contracts increased 8.7 percent to 3,270, while food service contracts increased 7.4 percent to 2,065 [1]. Frost & Sullivan [2] found that the European healthcare IT outsourcing market generated revenues of US$396.4 million in 2005 and estimates they will reach US$697.7 million in 2010. Outsourcing is contracting with another company or person to do a particular function while off-shoring simply means having the outsourced business functions done in another country. Off-shoring is another type of outsourcing. Off-shoring is one situation in which developing countries that are able to adopt standards, processes, and language of developed countries can benefit from the liberalization of the movement of goods and services [3].

One of the strategic tools healthcare executives used to meet the cost-saving target is outsourcing. Even though outsourcing has many benefits, outsourcing will fail if not managed successfully. Hospital executives must choose outsourcing providers who hold the necessary leadership capabilities. Managing outsourcing requires an understanding of outsourcing strategy, the benefits and risks of outsourcing, the evaluation process, and the methods to managing outsourcing providers. With appropriate management, strategic outsourcing should provide healthcare executives with a viable strategy for controlling costs and maintaining quality patient care [4-6]. The most outsourced functions in healthcare are information technology (29 percent), finance (20 percent), and support services (19 percent) [7]. By outsourcing, hospitals can reap the benefits of medical device reprocessing without assuming additional staffing and compliance burdens. Outsourcing enables hospitals to implement a medical device reprocessing program quickly, with no capital investment and minimal effort [8]. Before negotiating any outsourcing transaction, hospital executives should carefully analyze the legal and regulatory implications, which will vary according to the type of services and the vendor involved [9].

Hodge [10] estimated the average cost savings, after including 2% for the cost of the contracting process, to be around 6–12%. In some cases, lower bids may not mean additional savings for the outsourcer because sometimes the apparent cost of delivering a service may not represent actual cost. For example, savings from low – cost wages may not compensate for the costs incurred from turnover and quality problems that come from an inexperienced, poorly trained, and unstable workforce [11,12]. apparent cost means the cost can be seen in the income statement for buy the service or goods. Actual cost means the cost other than in the income statement such as buy a poor quality of goods or services will occurs other cost in aftersale service or fix the good.

Young [13] stated that outsourcing resulted in increased staff morale, upgraded capital equipment and improved services [13]. The outsourcing of pathology and dental technical services aimed to increase labor flexibility, thereby decreasing costs. The outsourcing of lawn mowing was simply to reduce costs. However, food services in hospitals were not outsourced because there was a lack of evidence that costs could be reduced.

However, not all of the outsourcing is beneficiary to hospitals. Negative perceptions about the contract management groups of emergency department outsourcing persist among some physicians [14]. Also, the contracting out of the orderly/porter/courier service at Sir Charles Gairdner Hospital in Western Australia shows negative results. The result was poor in terms of cost, quality and externalities [15]. Guy [16] suggested that hospitals should be wary of common myths that can cause them to make missteps in developing clinical service outsourcing.

Contracting out also can be a relatively cost-effective way to cut 13–17% cost of the total prevention budget in Africa [17]. Liu [18] suggested that contracting-out has improved access to services.

Within the health sector and the Human Services Department, the Australian government instructed clinical and non-clinical areas to be market tested through benchmarking services against the private sector, with the possibility of outsourcing. These services included car parking, computing, laundry, engineering, cleaning, catering, medical imaging (radiology), pathology, pharmacy, allied health and general practice. Managers, when they choose between outsourcing, and internal servicing and production, would thus ideally base their decision on economic principles [13].

The March 2003 severe acute respiratory syndrome (SARS) outbreak from China [19] to Taiwan has had an obvious impact on local hospitals' outsourcing ability [20]. Dr. Su [21], Director of Center of Disease Control in Taiwan, ordered the shutdown of the outsourcing system due to the rapid transmission of the virus through hospitals by the carelessness of the housekeeping and laundry services during SARS break.

### Objectives

This study explored outsourcing in Taiwanese hospitals, comparing the differences between them based on hospital ownership and accreditation level. This paper also assesses the degree of hospital satisfaction with the outsourcing of medical and non-medical items and conduct factor analysis on satisfaction factors.

## Methods

### Setting

The research samples are from the hospital data from Taiwan's 2005 to 2007 Department of Health qualifying lists of accreditation. According to Taiwan's system, the hospitals were accredited into three levels: medical center, region, and local. Facilities that are categorized as "medial centers" have more than 800 beds and are affiliated with a medical school.

### Design

This research combined two kinds of methods: a questionnaire survey and the in-depth interview to CEOs of the two sample hospitals. For in-depth interview, one hospital is not-for-profit and located in central Taiwan, while the other is a public hospital located in Taipei. Two of the hospitals assessed were medical centers with more than 1000 general beds and they were well known on outsourcing in medical items as well as on the non medical items. The in-depth interview were to ask the hospital's situation about outsourcing, and the feeling include what they think about the outsourcing can get benefit to them and the advantage and disadvantage of it. The interview time lasted for continuously four hours. The questionnaires were mailed to all 17 medial centers and all 71 region hospitals, while the 77 local hospitals were chosen by stratified system sampling methods from 382 local hospitals. The sample medical centers have the bed number from 800 to 2500, region hospitals' size normally between 300 beds to 800 beds, and local hospitals are from 20 to 300 beds in Taiwan. The total sample size is 165, with 37 completed questionnaires returned; The returned samples in medical centers were 6/14 = 35.3%, in regional hospitals were 21/71 = 29.5%, and in local hospitals were 10/77 = 12.9%. We prudently analyzed the returning samples and find they were evenly spread across the north, central, and south region of Taiwan; therefore, we believe it can represent the Taiwan hospital system. However, the returned local hospital samples were few, the reason could be: this level of small hospitals always own and operate by physicians and his/her families, therefore, unwilling to fill this kind of academic questionnaire due to lack of manpower.

The returned questionnaires were analyzed with STATISTICA^® ^7.1 version. The Pearson chi-square, ML chi-square, paired t test, factor analysis and structure equation modeling (SEM) were used to get the statistical results.

### Instruments

The full questionnaire was listed on Appendix (see Additional file 1) and the design combined the research of Kirchheimer, Shinkman, Martanegara, and Yigit [1,7,22,23]. The questionnaire used in this study was a semi-structured questionnaire, composed of five parts. The first part consists of the questions regarding the title and reasons to conducting this research. The second part is the hospital accreditation level, such as medical center, regional, or local; total hospital beds; and hospital type, such as public, private, or not-for-profit. The third part is the definition of outsourcing, and inquires whether the hospital outsources at 9 non-clinical items and 10 clinical items which categories revised from Yigit (2007) paper. The fourth part is the satisfaction scores, using Likert 5 scales to evaluate the outsourcing of medical and non-medical items. The fifth part is the hospital's comments or suggestions.

### Validity

#### Content validity

Content validity of the questionnaire was further confirmed by 3 directors of medical doctor and 2 hospital management specialists. The validity was also verified by several literature reviews on the questionnaire, which include Kirchheimer, Shinkman, Martanegara, and Yigit [1,7,22,23].

#### Construct validity

An exploratory factor analysis was conducted on the non medical items and medical items satisfaction scores to further examine the dimensionality of the scale.

This program been proved from the ethical committee: Biomedical Research Center, CSMU, Taiwan within Document CSMU-BMRC-97-001.

## Results

This research conducted the scale's reliability, and underlying dimensionality.

### Reliability

#### Internal consistency reliability

The reliability of the fourth part of the questionnaires showed the Cronbach's α = 0.820. None of the item-to-total correlation for the individual satisfaction items was less than the 0.35 cut-off value [24].

The results of the second part of the questionnaires showed in Table 1, in hospital type, the private hospitals are smaller in size and without medical center, while the not-for-profit hospitals are much larger. None of them are considered local hospitals. For hospital level, five of the six medical centers are not-for-profit, and seven of the eleven local hospitals are private.

**Table 1:** Sample Hospitals–Hospital Level and Hospital Type

Level	Type: Private	Type: Not-for Profit	Type: Public	Row Totals
Local Hospital	7	0	3	10
Region Hospital	4	11	6	21
Medical Center	0	5	1	6

All Groups	11	16	10	37

The results of the third part of the questionnaires are shown in Table 2. For non-medical items: common and medical waste has the highest percentage (94.6%) of outsourcing. The gift store (75%) and linen (73%) follow that high. The lowest percentage is in utility maintenance (13.5%). The reason that the medical and common waste outsourcing rate is high is because Taiwanese hospitals have limited space, and lack the area to build refuse burning facilities. Similarly, the newer hospitals are not built with laundry facilities because the laborers and machines take up too much space. Outsourcing this service has saved hospitals space and funds. The gift store is also frequently outsourced because it is not one of the hospital's major concerns; therefore, they usually rent the space out or cooperate with chains of retail stores.

**Table 2:** Rate of Outsourcing for Taiwanese Hospitals

Non-Medical Items	Number	Outsourced number	Outsourced Percentage (%)
Medical Waste	37	35	94.6
Common Waste	37	35	94.6
Gift Store	37	28	75.6
Linen	37	27	73.0
Restaurant	37	21	56.8
Security Guard	37	20	54.1
Information	37	18	48.6
Medical instrument Maintenance	37	14	37.8
Utility Maintenance	37	5	13.5

Medical Items	Number	Outsourced number	Outsourced Percentage (%)

Ambulance	37	19	51.4
Hemodialysis	34	17	50.0
Laser	32	8	25.0
Shake Wave	31	7	22.6
Laboratory	37	5	13.5
Radiology	37	4	10.8
Health Exam	37	4	10.8
Nutrition	34	1	2.9
Nurse	37	1	2.7
Pharmacy	37	1	2.7

For medical items, the highest rate of outsourcing is in the ambulance department (51.4%). A hospital chief of the executive officer (CEO) expressed that night shift and holiday shift ambulances are always contracted out due to the higher cost and the limited human resource concern. The hemodialysis center also has a higher rate of outsourcing (50%) due to there are four giant hemodialysis medical group in Taiwan that hire physicians, nurses, technicians, and purchase discounted hemodialysis filters and supplies. In general, these hemodialysis companies could provide unify and better quality with a reasonable price. However, the other medical items show a much lower percentage of outsourcing rate such as nutrition, pharmacy, and nursing, the outsourcing rate is lower than 3%. This shows that hospitals consider medical items are their core business and very conservative in outsourcing medical items.

The outsourcing results also test whether the hospital type and level are associated with tendency to outsource. The Pearson Chi-square tests and ML Chi-square were performed and recorded on Table 3. In the hospital type factor, utility maintenance and security guard services are statistically significant at the alpha equals 0.05 level. Both items are assessed from not-for profit hospitals that have higher outsourcing percentage levels than private and public hospitals. In the hospital level factor, two items are significant: the gift store and ambulance service. Between them, ambulance services have a higher outsourcing percentage in the regional hospitals while the gift store percentages are higher in regional hospitals as well as in medical centers.

**Table 3:** Summary of Chi-square Tests that were Significant

By Hospital Level	Pearson Chi-square	ML Chi-square
Non Medical – Gift Store	9.60(p = .008)	8.98(p = .011)
Medical – Ambulance	8.10(p = .017)	8.51(p = .014)

By Hospital Type	Pearson Chi-square	ML Chi-square

Non Medical – Utility Maintenance	7.56(p = .023)	9.43(p = .009)
Non Medical – Security Guard	8.74(p = .013)	9.26(p = .010)

Remark: testing alpha = .05

The results of the satisfaction scores on the fourth part of the questionnaire are listed on Table 4. A paired t-test was conducted between medical items and non-medical items. In non-medical items, the higher scores is saving human resource (HR, 3.92) while the service quality (SQ) and saving capital investment (SI) got the lowest score (3.03). In medical items, the speciality performance (SP) and SQ have a higher score (4.16) and saving cost (SC) has the lower score (3.38). The paired t-test between medical and non-medical items shows only the HR has no statistic significance; other items such SP, SQ, SI, adaptability to environment (AE) and total satisfaction (TS), medical items have statistic higher score than non-medical items. Only in the cost saving do the non-medical items have a higher score than the medical items. In general, the medical items have higher scores than the non-medical items. The reason that medical items can surpass the non medical items on satisfaction score is the medical items need more professional training, purchase and maintain medical equipment, and normally the size of the outsourcing companies are much bigger and show their companies' discipline and speciality performance on their job. However, the "saving cost" shows different results, this could be the outsourcing of the non-medical items such as linen and maintenance did pay less to outsourcing companies.

**Table 4:** Summary for Satisfaction with Medical Items, Non-medical Items, and Paired t-Test

Survey Item	M_non_	M	SD_non_	SD	t-Value	p-Value
speciality performance	3.24	4.16	.723	.602	7.03	0.000*
service quality	3.03	4.16	.726	.553	7.52	0.000*
saving capital investment	3.03	4.08	.687	.640	8.22	0.000*
saving cost	3.59	3.38	.725	.828	-2.09	0.044*
saving human resource	3.92	3.86	.682	.822	-0.47	0.644
adaptability to environment	3.62	4.00	.721	.745	3.87	0.000*
total satisfaction	3.49	3.84	.692	.602	2.84	0.007*

N = 37. Remark: alpha = .05, "*" means statistical significant

### Dimensionality

To examine the dimensionality of the scale, an exploratory factor analysis was conducted on the non medical items and medical items satisfaction scores. Factor analysis results are listed in Table 5. When subjected to oblique rotation, and combine with the Scree plot of Eigenvalue, the loading provide a three meaningful factor structure on non medical items and two factors on medical items. The interesting point is that the "saving cost" is significant on both factor 1 and factor 2 of non medical items. For non medical items, this study define the first factor is "performance", this means hospitals believe outsourcing have better ability on the performance. The second factor is "finance", this means outsourcing can contribute hospital's finance and also help hospitals to overcome the dramatically changing medical environment, such as the keeping going down reimbursement system of the "individual hospital global budget" of National Health Insurance of Taiwan. The third factor is "human resource", such as the security, linen, and waste treatment, outsourcing have great contribution to hospital's limited human power. For medical items, this study defined the first factor is "operation" since this factor include most of the hospital's operation indices, and second factor could be defined as "satisfaction".

**Table 5:** Exploratory factor analysis (N = 37)

	Factor loadings		
	
Items	Factor 1	Factor 2	Factor 3
N-speciality performance	0.73		
N-service quality	0.76		
N-saving capital investment		0.73	
N-saving cost	0.61	0.63	
N-saving human resource			0.91
N-adaptability to environment		0.87	
N-total satisfaction	0.65		

Eigenvalue	2.33	1.44	1.04
Variance explained	27.84%	24.38%	16.47%

speciality performance	0.71		
service quality	0.67		
saving capital investment	0.75		
saving cost	0.63		
saving human resource	0.66		
adaptability to environment		0.84	
total satisfaction		0.69	

Eigenvalue	2.65	1.26	
Variance explained	33.41%	22.50%	

* Significant at the 0.60 level

**Figure 1:**
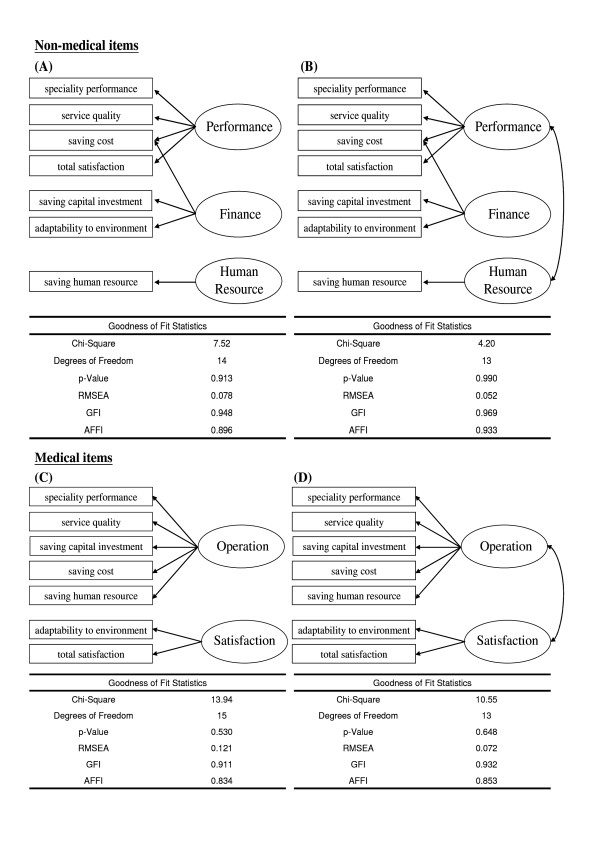
**Confirmative Factor Analysis results of non-medical items and medical items**.

To further exam the factor validity and reliability of the satisfaction model, a confirmative factor analysis (CFA) was conducted using structure equation modeling (SEM) method [25] and the results showed on Figure 1.

To assess adequate fit of the model, Hoyle have recommended several indices the chi square (χ^2^) test, P value, the goodness-of-fit index (GFI), AGFI, and the root mean square error of approximation (RMSEA) [26]. The P value greater than 0.05, and the values as close as possible to 0.9 are recommended for the GFI and AGFI and the values below 0.08 for the RMSEA are recommended [27,28]. For non-medical items, the initial model is Figure 1A, the model indicating and acceptable model of it. After several times model modifications include connect the relationship between the three latent variables, the other model on Figure 1B shows an even better result. Therefore, the Figure 1B is our final model and the correlations between two latent variables "performance" and human resource" is significant. For medical items, the Figure 1D model also show a better model fitting than Figure 1D and the correlation between "operation" and "satisfaction" is significant.

Among the 37 effective answers, 12 of which wrote comments about the outsourcing and listed as follows:

1. Advantage of outsourcing for public hospitals:

Full-time-equivalent (FTE) concern: Due to government regulations, public hospitals have limited FTE for each hospital, and outsourcing helps hospitals to get more FTE without hiring workers themselves.

2. Concerns in outsourcing:

i. Hospitals can easily contract out for restaurants and gift shops, but much consideration is needed for the contracting of vital items such as a multi-detector CT scan.

ii. Hospitals have to set up a system or director to review and monitor the implementation of outsourcing companies, and if they do not comply with the contract, consequences must be made.

iii. Epidemiology concern: During the 2003 outbreak of SARS, outsourcing workers, such as the housekeepers, were the primary media of virus transmission. Their lack of training and carelessness contributed greatly to the perpetuation of the disease.

3. Disadvantages of outsourcing:

i. When hospitals encounter malpractice or law suits from patients it is not easy to identify the responsibility.

ii. The outsourcing companies provide hospitals with cutting edge information and technology. If they cease contract suddenly, hospitals lose vital items that are required for proper function.

iii. The contract documents are not easy to fill out, and are based on honesty, equality, and mutually trust.

Results of the in-depth interview with the CEOs of two hospitals:

1. Determination of which items can and can not be outsourced: The decision differs between public and private hospitals. In not-for-profit hospitals, the CEO stated that only the CEO, CFO, and the Director of the Finance can make the choice to outsource an item. The public hospital's CEO however, is more conservative, and stated that unless the government permits a deviation, it is better to comply with the declared laws and regulations.

2. Advantage of outsourcing of in two kinds of hospitals:

i. Saves the energy of management personnel: outsourcing contractors can help manage a large staff.

ii. Improves efficiency and employee morale: in some departments, such as the physical therapy, the working hours were extended from two shifts to three, and the workers received 60 to 100 percent more salary after outsourcing.

iii. Labor regulation consideration: Taiwan implemented strict labor laws in 1998, requiring employers to pay more to the retirement beneficiary.

iv. Capital consideration: the rate of upgrade in medical instruments runs too high for hospitals to afford. Outsourcing has helped hospitals acquire new instruments without financial burden.

v. Improved services: outsourcing services are flexible in recruitment, and offer better training programs and wages, which result in better service to customers.

3. Advantage of outsourcing for public hospitals:

Outsourcing helps public hospitals to get more FTE without hiring workers themselves.

4. Disadvantage of outsourcing for public hospitals:

Due to regulations, the public hospitals have to use bids to select outsourcing companies. Although the necessity of some items is measured by quality, cost is always the key indicator of purchase. Also, when the contract is up (normally one to three years), hospitals are required to bid again, making it difficult for the outsourcing companies to consistently provide high quality service.

## Discussion

Compared with Shinkman's [7] study, this study shows higher outsourcing percentage in information (48.6% vs. 29%). One of the reasons is that Taiwan's software industry is powerful and can provide strong support to hospitals. The other reason is that due to Taiwan's single payment system of National Health Insurance, software companies can almost uniformly copy software systems to contract hospitals with the quality and lower cost under today's open mainframe computer system.

The results of this research also showed that the outsourcing of common and medical waste is a very high percentage (94.6%). On the other hand, the outsourcing of nutrition, nursing, and pharmacy have a lower percentage (less than 3%). Hospitals, especially for public hospitals, can get benefits from outsourcing to revive the FTE and human resource limitation. These results are the same as the Robert, Quinn, Jennings, and Yang [4-6,13]. However, the drawbacks of regulations have limited the public hospitals to operate the outsourcing contracts. This result is somewhat like the Boardman [15].

Compare with the Moschuris's [29] study, this study showed the same in the main factors affecting outsourcing decision. However, this study have the lower scores in saving cost (SC) is unlike Moschuris's results.

According to results from Table 4 and the other results of this study, we suggest hospitals pay more attention to the service quality of non-medical, outsourced items. Hospitals must also maximize financial and human resources advantage by outsourcing services such as laundry facilities, gift stores, and information as often as possible. In general, hospitals have higher satisfaction scores in medical items than in non-medical items.

Although outsourcing should provide healthcare executives with a viable strategy for controlling costs, reduce administrative hassles, and maintaining quality patient care, hospitals should be wary of common myths that can cause them to make missteps in developing clinical service outsourcing arrangements [16].

### Limitations

This limitation of this study is that the practical value can be generalized only to the hospitals system similar with Taiwanese health care.

## Conclusion

Hospitals, especially for public hospitals, can get benefits from outsourcing to revive the full-time-equivalent and human resource limitation. Other advantage such as save the energy of management personnel, improves efficiency and employee morale, and help hospitals acquire new instruments without financial burden.

## Competing interests

The authors declare that they have no competing interests.

## Authors' contributions

CTH was responsible for primary data cleaning and analysis, JYP was responsible for primary study design, manuscript drafting, statistic and interpretation, and manuscript submission. HC served as a methodologic consultant, assisted with data analysis and interpretation, and participated in manuscript editing.

## Pre-publication history

The pre-publication history for this paper can be accessed here:



## References

[B1] Kirchheimer, Barbara (2005). Outsourcing ins and outs. Mod Healthc.

[B2] Frost, Sullivan (2006). Reports European healthcare IT outsourcing market to offer lucrative opportunities. Hosp Bus Week.

[B3] Christophe S, Brain H, Pierre-Henri B (2005). Globalization in health care: is international standardization of quality a step toward outsourcing. Int J Qual Health Care.

[B4] Roberts, Velma (2001). Managing strategic outsourcing in the healthcare industry. J Healthc Manag.

[B5] Quinn JB (2000). Outsourcing innovation: the new engine of growth. SMR.

[B6] Jennings D (1998). Strategic guidelines for outsourcing decisions. Strategic Change.

[B7] Shinkman R (2000). Outsourcing on the Upswing. Mod Healthc.

[B8] Haley, Deborah (2004). A case for outsourcing medical device reprocessing. AORN Journal.

[B9] Callahan John M (2005). 10 practical tips for successful outsourcing. Healthc Financ Manag.

[B10] Hodge GA (2000). Privatization an international review of performance.

[B11] Mobley M (2000). What You Need to Know About Outsourcing HR Functions. HR Focus.

[B12] Allen S (2000). Outsourcing Services: The Contract Is Just the Beginning. Bus Horiz.

[B13] Young SH (2003). Outsourcing and benchmarking in a rural public hospital: does economic theory provide the complete answer?. Rural Rem Health.

[B14] Meyers S (2004). ED Outsourcing: Is it good for patient care?. Trustee.

[B15] Boardman AE, Hewitt ES (2004). Problems with contracting out government services: lessons from orderly services at SCGH. Ind Corp Change.

[B16] Guy RA, Hill JR (2007). 10 outsourcing myths that raise your risk: hospitals should be wary of common myths that can cause them to make missteps in developing clinical service outsourcing arrangements. Healthc Financ Manag.

[B17] Marek T (1999). Successful contracting of prevention services: fighting malnutrition in Senegal and Madagascar. Health Policy and Planning.

[B18] Liu X, Hotchkiss DR, Bose S (2008). The effectiveness of contracting-out primary health care services in developing countries: a review of the evidence. Health Policy and Planning.

[B19] Hu G, Rao K, Sun Z, Sun Z (2007). An investigation into local government plans for public health emergencies in China. Health Policy and Planning.

[B20] Ksiazek TG, Goldsmith CS, Zaki SR (2003). A Novel Coronavirus Associated with Severe Acute Respiratory Syndrome. NEJM.

[B21] Su IZ "The outsourcing system of the hospitals should be shutdown, lesson from Taiwan SARS outbreak". 2003. SARS/The lesson from the epidemic situation. Director of CDC of Taiwan.

[B22] Martanegara V, Kleiner BH (2003). Effective employment screening in the American health care industry. Manag Res News.

[B23] Yigit V (2007). Outsourcing and its implications for hospital organizations in Turkey. J Health Care Finance.

[B24] Nunnally CJ (1978). Psychometric Theory.

[B25] Chang HH, Chang CS (2008). An assessment of technology-based service encounters & network security on the e-health care systems of medical centers in Taiwan. BMC Health Serv Res.

[B26] Hoyle RH, Panter AT, Hoyle RH (1995). Writing about structural equation models. Structural equation modeling: concepts, issue, and applications.

[B27] Browne MW, Cudek R, Bollen KA, Long JS (1993). Alternative ways of assessing model fit. Testing structural equation models.

[B28] Schutz RW, Duda J (1998). Assessing the stability of psychological traits and measures. Advances in sport and exercise psychology measurement.

[B29] Moschuris SJ, Kondylis MN (2006). Outsourcing in public hospitals: a Greek perspective. J Health Organ Manag.

